# Effect of Silver Particle Distribution in a Carbon Nanocomposite Interlayer on Lithium Plating in Anode-Free All-Solid-State Batteries

**DOI:** 10.1021/acsami.5c06550

**Published:** 2025-06-26

**Authors:** Michael Metzler, Christopher Doerrer, Yige Sun, Guillaume Matthews, Enzo Liotti, Patrick S. Grant

**Affiliations:** † Department of Materials, 98592 University of Oxford, Parks Road, Oxford OX1 3PH, U.K.; ‡ The Faraday Institution, 6396 University of Oxford, Quad One, Becquerel Ave, Harwell Campus, Didcot OX11 0RA, U.K.; ¶ Department of Mechanical and Industrial Engineering, University of Toronto, 7938 University of Toronto, 5 King’s College Road, Toronto, ON M5S 3G8, Canada

**Keywords:** spray printing, anode-free, silver−carbon layer, secondary-ion mass spectrometry, solid-state battery manufacturing, battery structural design, layer-by-layer

## Abstract

Solid-state batteries can outperform lithium-ion batteries in energy per unit mass or volume when operating with a Li metal anode. However, Li anodes pose significant manufacturing challenges. Anode-free cells avoid these challenges by plating metallic Li at the anode on the first charge, but subsequent nonuniform cyclic stripping and plating decrease the Coulombic efficiency and encourage Li dendrites and early cell failure. We report a new spray-printed nanocomposite bilayer of silver/carbon black (Ag/CB) between anodic current collectors and a Li_6_PS_5_Cl solid electrolyte comprising an Ag-rich region at the current collector and a CB-rich region at the solid electrolyte. Compared with previous Ag/CB mixtures, this bilayer promoted more uniform Li anode plating and improved cycling. Cells with a high-Ni oxide cathode had an initial discharge capacity of >190 mAh/g and a Coulombic efficiency of >98% over 100 cycles. Improved Li plating uniformity with the structured Ag/CB interlayer was confirmed by using secondary-ion mass spectrometry (SIMS) imaging.

## Graphical abstract

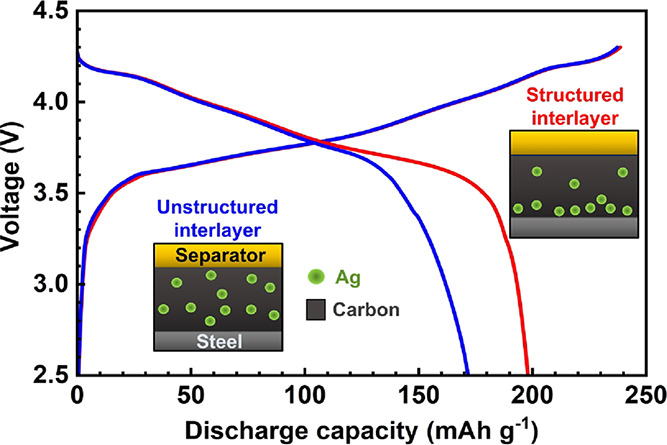

## Introduction

Lithium-ion batteries (LIBs) using liquid organic electrolytes are the dominant technology for electrochemical energy storage. However, LIB technology is reaching a plateau of energy density at approximately 270 Wh/kg and 650 Wh/L, principally related to the intrinsic capacity of the anode and cathode active materials.^1,2^ LIBs also rely on toxic and flammable organic electrolytes that provide persistent challenges to cost, safety, and environmental compatibility. Solid-state batteries (SSBs) use a more chemically inert solid-state electrolyte (SSE) that allows the use of a Li metal anode with a high theoretical energy density of 3860 mAh/g.^3,4^ SSEs operate at a relatively high negative potential (−3.06 vs NHE) that increases battery energy density up to ≥400 Wh/kg and ≥1000 Wh/L.^5,6^

However, Li metal anodes in SSBs pose significant manufacturing challenges. Li foils (3–50 μm) are fabricated by extrusion/rolling but have high reactivity with air and stickiness to fabrication tools.^7,8^ Li (1–10 μm) can be deposited by physical vapor deposition (PVD), but it is slow and cost-intensive.^9^ Electrochemical Li deposition using a galvanic bath is also slow, with high costs for electrolyte regeneration and disposal.^10^ Alternatively, plating the Li anode in situ from a Li-rich cathode on the first charge cycle simplifies battery assembly significantly, reduces material and production energy consumption, and avoids handling large-area Li foils/coatings.^11–13^ Critical to success is ensuring that Li plates homogeneously at the anodic current collector on the first and subsequent charge cycles, i.e., with constant thickness over all the current-collector area. To facilitate plating homogeneity, a thin layer (tens of μm) of a mixture of fine Ag (∼60 nm) and carbon black (CB, ∼35 nm) particulate can be used.^14–18^ Upon subsequent discharge, the Li anode is stripped entirely, and only the Ag/CB interlayer remains.^19^ If Li plating/deposition is not controlled, preferential Li deposition at defect sites, surface asperities, etc leads to inhomogeneous Li deposition and filamentary Li metal growth termed a “dendrite”.^20–22^ Inhomogeneous plating forms voids that reduce SSE/Li contact area and so increase local current densities, further encouraging Li dendrites.^23,24^

The mechanism by which the Ag/C layer homogenizes Li plating is an area of ongoing research, but for an Ag/graphite interlayer at 1 mA/cm^2^, Li first intercalated into graphite, followed by reaction of Li-intercalated graphite with Ag to form Li-rich phases, and finally Li–Ag alloy precipitated on the current collector. At >2 mA/cm^2^, direct Li metal formation was suggested.^25^ The role of the Ag nanoparticles is to facilitate uniform Li and Li–Ag deposition compared with carbon-only interlayers, but Ag does not itself affect the critical current density for dendrite formation. Because Ag and Li have some mutual solubility, it has been conjectured that Ag particles reduce the nucleation energy for Li formation, which manifests as a reduction in the nucleation overpotential for plating.^26–28^ The fine-scale C particulate surrounding the Ag nanoparticles also provides a high specific surface area for Ag/Li reactions.^29^ Other metal particles can also catalyze Li plating but require higher overpotential.^30^

We used spray printing to deposit composite Ag/CB interlayers on an SSB anodic current collector, without and with a concentration of Ag particles at the current collector. The hypothesis is that improved Li plating and stripping performance will be realized by encouraging Li plating specifically onto the current collector, which has not previously been considered. Spray printing allows large-area and microstructure layering unavailable by conventional processes such as slurry casting.^31^ For the unambiguous identification of plated Li, we used plasma-focused ion beam scanning electron microscopy (P-FIB SEM) combined simultaneously with energy-dispersive X-ray spectroscopy (EDS) and secondary ion mass spectroscopy (SIMS).

## Results and Discussion

The manufacture of Ag/CB interlayers and our SSB cell design is illustrated in Figure 1. A slurry of Ag nanoparticles (60 nm) and CB particles (80 nm) dispersed in a mixture of 100 mL of isopropyl alcohol (95 vol %)/*N*-methylpyrrolidone (5 vol %) containing polyvinylidene fluoride (PVDF) binder was spray-printed onto a 10 μm-thick stainless steel foil anodic current collector. The composite interlayer had a mass of 277.9 mg and comprised 22.5 wt % Ag nanoparticles, 67.5 wt % CB, and 10 wt % PVDF binder. The total printed area was 10 × 10 cm^2^, and the as-sprayed interlayer thickness was 20 μm. For random Ag/CB mixtures, termed “unstructured interlayers”, the Ag:CB weight ratio was 1:3, whereas for interlayers enriched in Ag in the region next to the current collector, termed “structured interlayers”, the same overall Ag fraction was divided into two separate solutions. The first deposited solution contained 87.5% of the Ag, and the second solution contained the remaining 12.5%, representing C:Ag weight ratios of 1:13 and 1:1, respectively, resulting in an Ag-rich and Ag-deficient bilayer arrangement. From the coated foil, 0.19 cm^2^ circular discs were laser cut (Figure 1b) with no interlayer debonding typical of mechanical punching (Figure S1). The discs were assembled as anodic current collectors in the SSB arrangement depicted in Figure 1c.

**Figure 1:**
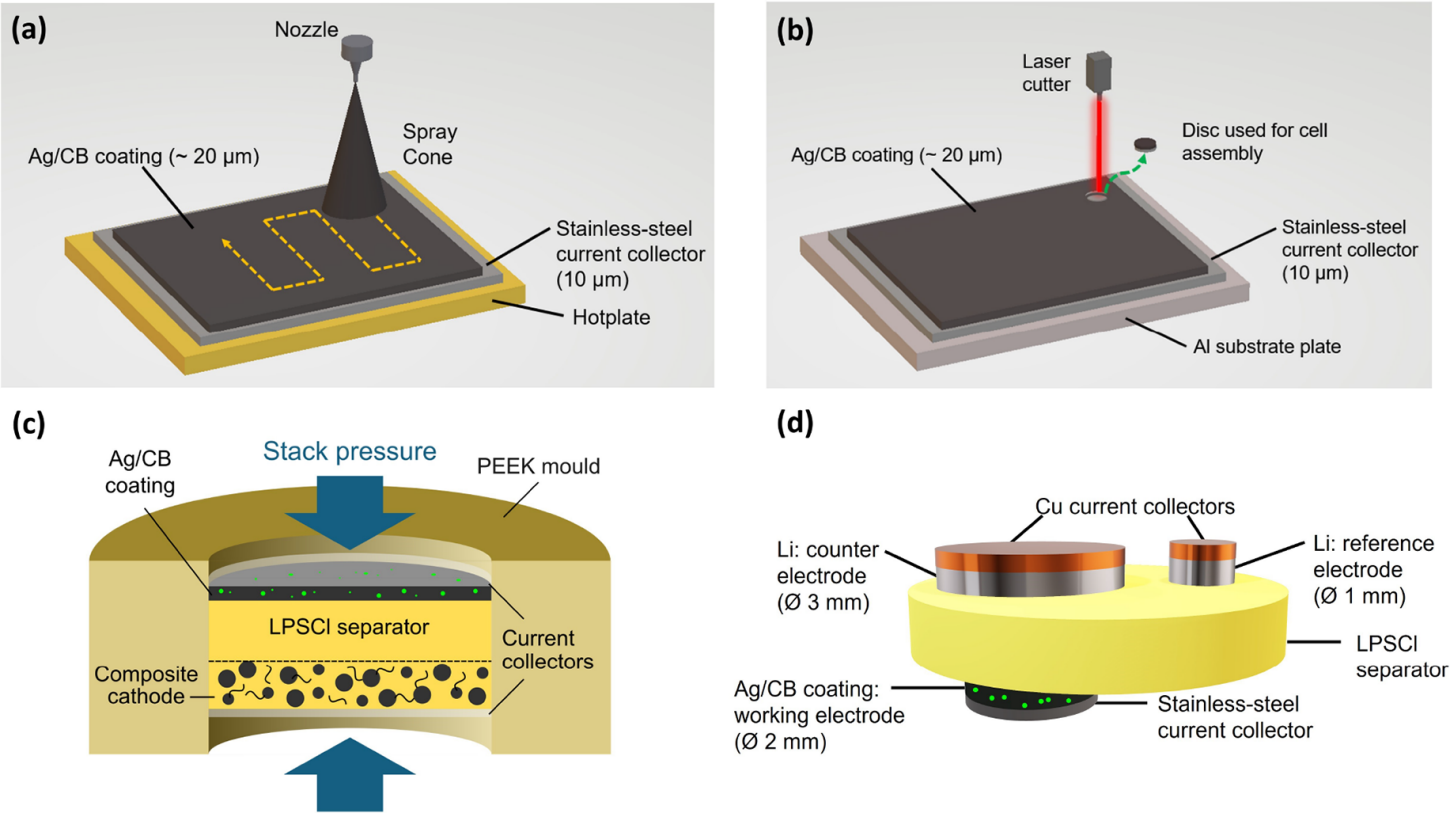
Summary of cell production. (a) Illustration of spray printing; (b) illustration of laser cutting; (c) illustration of the SSB full-cell arrangement; and (d) illustration of the three-electrode arrangement.

The resulting pellet-like SSB therefore comprised: Ag/CB interlayer, Li_6_PS_5_Cl (LPS) SSE separator, and a composite cathode consisting of a mixture of single-crystal LiNi_0.83_Mn_0.06_Co_0.11_O_2_ (NMC, 1–2 μm), LPS, and carbon nanofibers, with a total mass of 4 mg at an overall mass fraction ratio of 75:22:3. The assembly was uniaxially pressed at 500 MPa at room temperature to reduce porosity and ensure interfacial contact between the layers; the compressed interlayer thickness was ∼13 μm. The seminal paper of Lee et al.^19^ showed some of the best-performing cells with Ag/CB layers, which were prepared in a workflow that included warm isostatic pressing (WIP) of the cell assembly to promote density and interfacial contact. Here, we used the more common laboratory approach of uniaxial cold pressing of the separator and composite cathode, with a focus on the relative performance of unstructured and structured interlayers, rather than absolute performance. Cells were cycled at a uniaxial stack pressure of 2–20 MPa and 60 °C in a custom-designed device under Ar atmosphere to facilitate Li^+^ ion mobility and to differentiate efficiently the relative performance of separate interlayers.^32^ All capacities herein are expressed as a fraction of the NMC mass contained within the composite cathode. Three-electrode half-cells with Ag/CB as working electrode (ϕ 2 mm) and Li as counter (ϕ 3 mm) and reference electrodes (ϕ 1 mm) were also assembled (Figure 1d) to separate contributions of working and counter electrodes to the nucleation overpotential.^33^ Three-electrode cells were charged at 1 mA/cm^2^, 4 MPa, and 60 °C to a capacity of 1 mAh/cm^2^. Three-electrode cells for microscopy were assembled by hot pressing at 500 MPa and 200 °C, charged at 2.5 mA/cm^2^ to a capacity of 5 mAh/cm^2^ at 60 °C and 4 MPa, and then disassembled in a glovebox.

The feedstock Ag and CB particle size distributions in suspension are shown in Figure S2a,b with approximately log-normal distributions and mean diameters of ∼60 and ∼80 nm, respectively. Figure S2c shows dry Ag particles readily forming >1 μm agglomerates. Amorphous rather than crystalline carbon may be beneficial to promoting uniform Li plating.^34,35^ High-resolution transmission electron microscopy (HRTEM) and X-ray diffraction (XRD) of the CB particulates (Figure S3) suggested a predominantly amorphous structure with crystalline subdomains.

Figure 2 shows SEM images of the cross-section of the as-manufactured (a) unstructured and (b) structured Ag/CB interlayers. Interlayers were free of large pores or delamination, and as intended, there was a marked difference in Ag distribution. Figure[Fig fig2]c,d shows corresponding Ag (green) and C (blue) EDS maps, indicating an approximately uniform distribution of 2–4 μm Ag agglomerates and smaller particles in the unstructured interlayer, and a ∼5 μm thick Ag-rich sublayer against the current collector for the structured interlayer, confirmed by quantitative image analysis in Figure S4.

**Figure 2:**
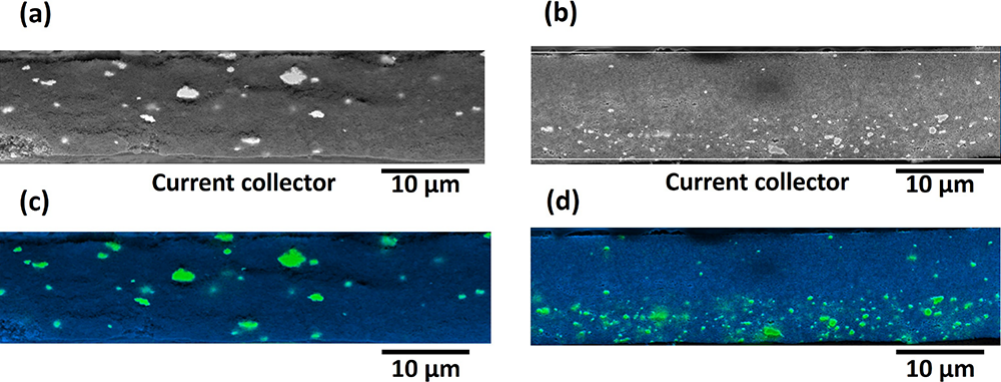
SEM cross-section images of the spray printed (a) unstructured and (b) structured Ag/CB interlayers after uniaxial compression at 500 MPa; corresponding superimposed Ag and C EDS maps of the (c) unstructured and (d) structured interlayers. Green: Ag, Blue: C.

Figure 3a shows a comparison of the first cycle voltage versus Li^+^/Li counter electrode as a function of time for the cell in Figure 1d and anodic current collector arrangements of stainless steel only and unstructured and structured Ag/CB interlayers. The sharp minima indicated the instant of Li plating.^36^ Stainless steel required the largest overpotential magnitude, with both unstructured and structured Ag/CB arrangements requiring ∼13 mV less overpotential.^37–39^ Figure S5 shows that there was no resolvable nucleation potential difference between unstructured and structured interlayers.

**Figure 3:**
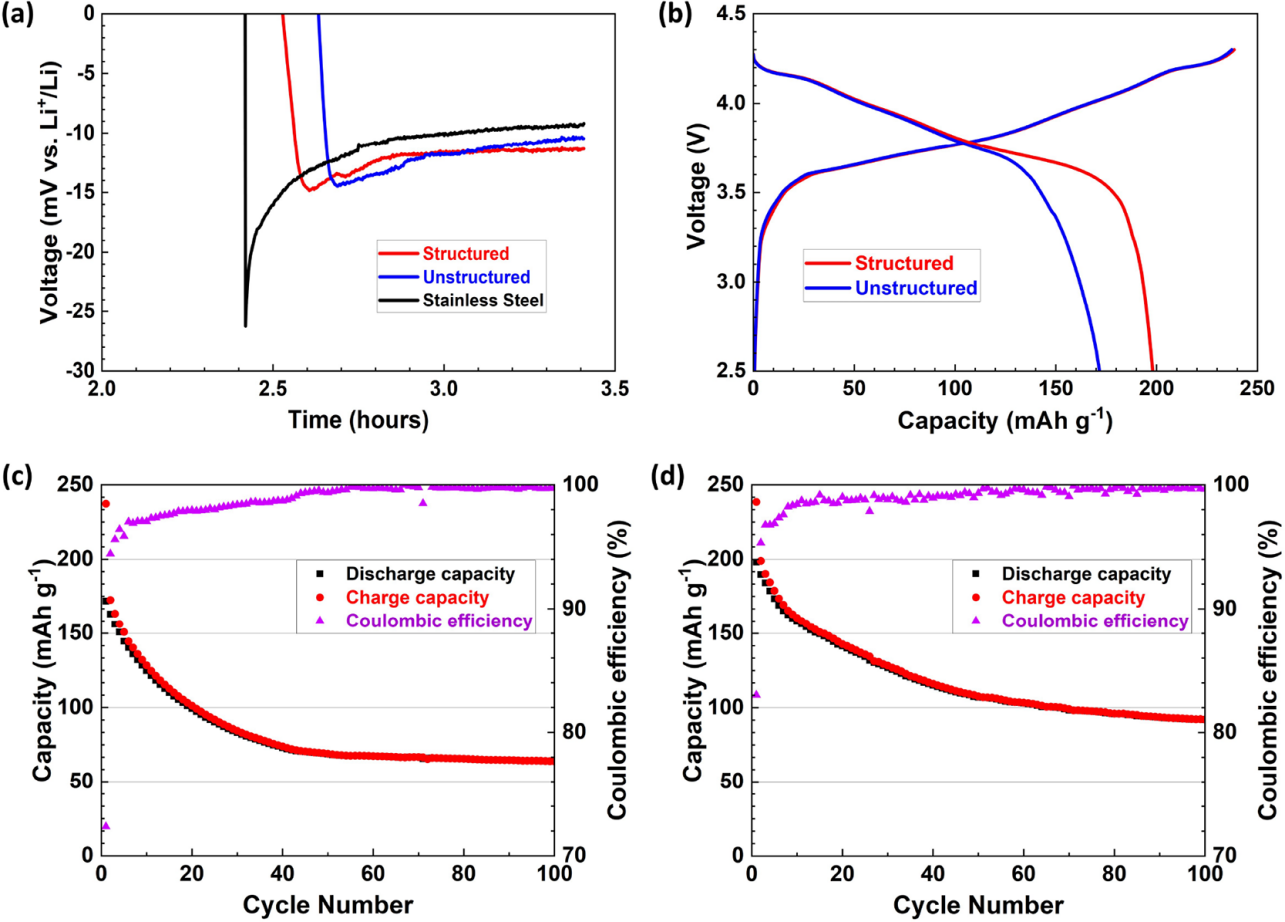
Cycling of SSB cells with Ag/CB interlayers at 60 °C and 4 MPa stack pressure. (a) Voltage/time during single-charge tests of three-electrode cells with and without Ag/CB interlayers at 1 mA/cm^2^; (b) Voltage/capacity during the first charge/discharge cycle of full-cells with unstructured and structured Ag/CB interlayers at 1 mA/cm^2^ charge/discharge current; Long-term full-cell cycling stability with an (c) unstructured and (d) structured Ag/CB interlayer at 1 mA/cm^2^ charge/discharge current.

Figure 3b shows the first cell charge–discharge cycle at a charge–discharge current of 1 mA/cm^2^ for the two Ag/CB interlayers. For the unstructured interlayer, the cathode discharge capacity was 172 mAh/g, which was comparable to 166 mAh/g for a similar arrangement.^40^ For the structured interlayer, the capacity increased to 198 mAh/g, and the initial Coulombic efficiency (CE) increased from 72 to 83%. Figure[Fig fig3]c,d shows the subsequent cycling stability at 1 mA/cm^2^. Both arrangements showed typical reductions in cathode capacity and no catastrophic failure, and CE > 99% after 100 cycles. The structured interlayer had improved charge and discharge capacity retention after 100 cycles, up from 27 to 39% and from 37 to 47%, respectively. In extended cycling, cell failure eventually occurs by Li dendrite growth through the separator because of the cumulative effect of Li plating inhomogeneity. When dendrites connect both electrodes electronically, an irreversible cell short occurs. Figure S6 shows the reproducibility of Ag/CB structuring on charge/discharge capacity for the first three cycles, with a systematic benefit of the structured interlayer greater than inevitable cell-to-cell variations.

For the sake of completeness, Figure S7 shows the inability of the uncoated stainless-steel current collector to provide any cycling response, and Figure S8 shows the Ag/CB interlayer effect at a lower discharge current density of 0.2 mA/cm^2^ and 20 MPa. Again, the structured interlayer provided an improvement in retained charge capacity, although the effect was less marked. Impedance analysis of full cells shown in Figure S9 revealed an ionic conductivity of ∼5 mS/cm and a stable response up to 10 cycles. Figure S10 shows improved capacity retention as a function of current density for structured interlayers, and failure of all cells at current densities >3 mA/cm^2^.

Figure 4a shows an FIB-SEM image of a cell cross-section, from the structured Ag/CB interlayer on the current collector region, after a single plating cycle at a relatively high current density of 2.5 mA/cm^2^. An approximately 20 μm thick layer had formed between the current collector (top of image) and the Ag/CB and the LPS separator (bottom of image). EDS maps for S, C, Ag, and Fe in Figure[Fig fig4]b–e unambiguously identify the stainless steel current collector (Fe-rich), LPS separator (S-rich), and Ag/CB layer (C, Ag-rich). However, EDS mapping could not unambiguously identify the new layer as Li because of the low X-ray energy of Li, the resulting low probability of X-ray emission, and the high likelihood of Li X-ray absorption.^41,42^ Therefore, SIMS was applied to the same region. In SIMS, a focused primary ion beam bombards the sample surface, sputtering secondary ions from the top few atomic layers that are analyzed on the basis of their mass-to-charge ratio in a mass spectrometer. SIMS can resolve light elements such as Li, but the signal intensity is not solely influenced by its local concentration and also depends on other factors such as material crystallography (known as the matrix effect), surface topography, etc.^43–45^ Notably, this effect can lead to Li intensity appearing higher for Li-containing compounds (such as LPS) than for metallic Li.^46,47^

**Figure 4:**
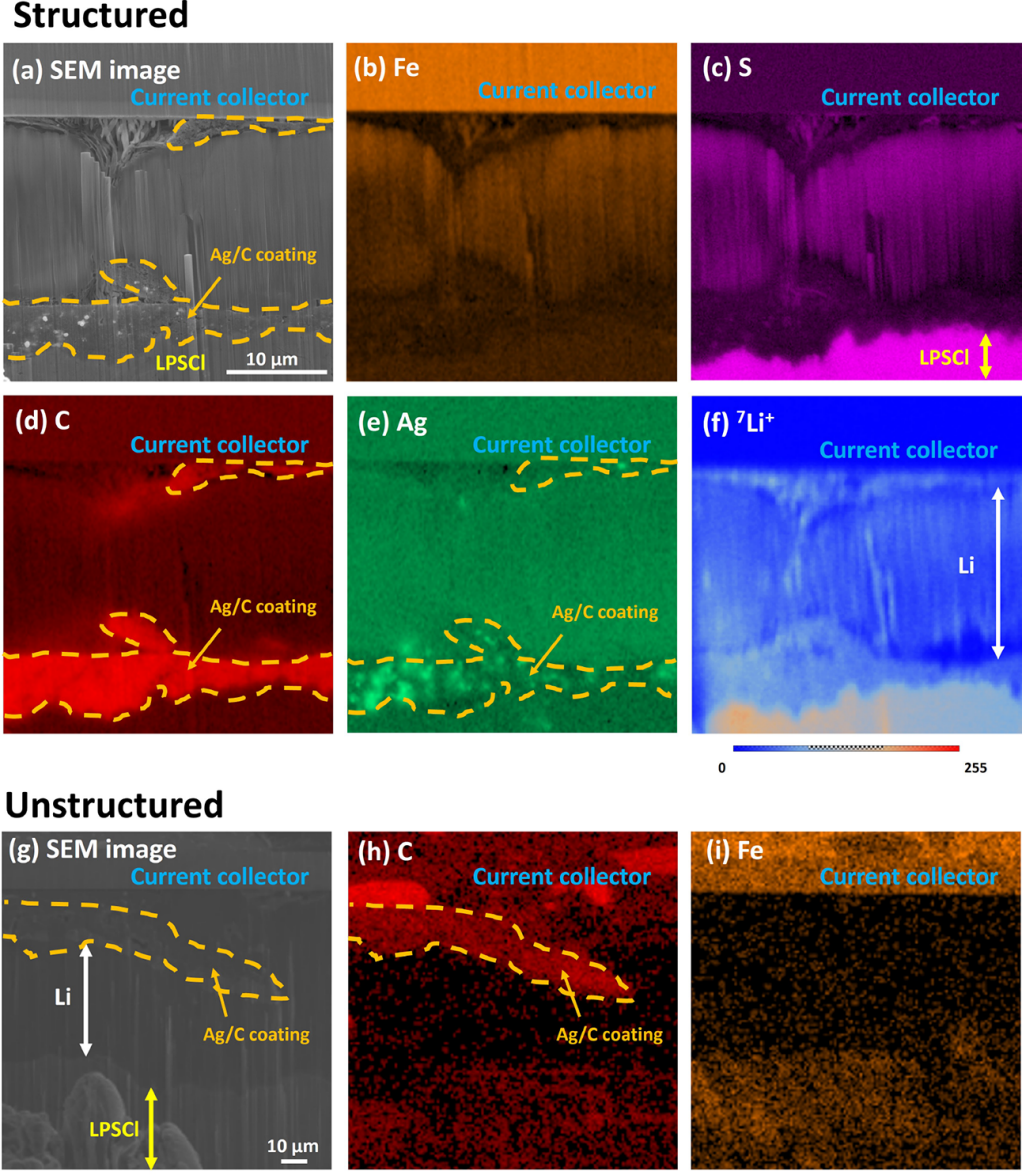
Cross-section of the stainless-steel current collector, deposited layer, Ag/CB interlayer, and LPS separator region for a single charge at 2.5 mA/cm^2^. (a) SEM image of the structured Ag/CB; (b–e) EDS maps of S, C, Ag, and Fe respectively; (f) ^7^Li^+^ SIMS map; (g) SEM image of the unstructured Ag/CB interlayer; and (h, i) EDS maps of C and Fe, respectively.

Figure 4f shows the SIMS Li^+^ map with the new layer adjacent to the current collector, yielding a strong Li signal sensitive to surface topography induced by the Xe^+^ milling. There was also a strong Li^+^ signal from the LPS separator. To assess if the Li signal from the layer could be consistent with metallic Li only and not another Li containing material, the relative intensities of the Li^+^ signal under the same SIMS imaging conditions used for a Li foil/LPS reference are shown in Figure S11. The Li:LPS Li^+^ intensity ratio from the reference was 38:100 and similar to 36:100 for the nominal Li layer and LPS separator in the sectioned cell. X-ray photoelectron spectroscopy (XPS) was also performed on the plated layer, as shown in Figure S12. Noting the difficulty in uncovering the layer while avoiding oxidation, the strongest intensity peak was due to oxidized Li metal. Overall, combining electrochemical, EDS, SIMS, and XPS investigations, the ∼20 μm layer in Figure 4 can confidently be ascribed to metallic Li plated on the first cell charge. The thickness was also consistent with approximate calculations of the expected thickness based on the recorded charge capacity (∼27 μm, see the Supporting Information).

Figure[Fig fig4]g–i shows a similar sequence of interlinked SEM images, C-EDS map, and Fe-EDS map from the anode region using an unstructured Ag/CB interlayer. Now, the Li layer formed predominantly between the Ag/CB interlayer and the LPS separator, increasing contact loss and impairing cycling behavior as shown in Figure 3.^48–52^ SEM images with a wider field of view and EDS maps of both interlayers after plating are shown in Figure S13, supporting the plating consistency benefits of the structured interlayer. Figure S14 shows a structured Ag/CB interlayer displaced away from the current collector by a uniform plated Li layer after charging at 2 mA/cm^2^. At an atomic level, the mechanism of Li intercalation, reaction with Ag, and Li precipitation and plating is very likely to follow a similar mechanism to that described by Spencer-Jolly et al.^25^ The difference from previous work is principally geometric due to the microstructural engineering of the interlayer that enhances the formation of Li directly onto the current collector rather than within and/or on the Ag/C interlayer.

Figure 5a summarizes schematically the effect of concentrating the Ag particles in the Ag/CB interlayer at the current collector. Initially, regardless of the Ag location, the Li^+^ ions are intercalated into the CB.^53^ As the CB becomes saturated, metallic Li is formed on and dissolves in the Ag particles due to their catalyzing effect in reducing the nucleation potential.

**Figure 5:**
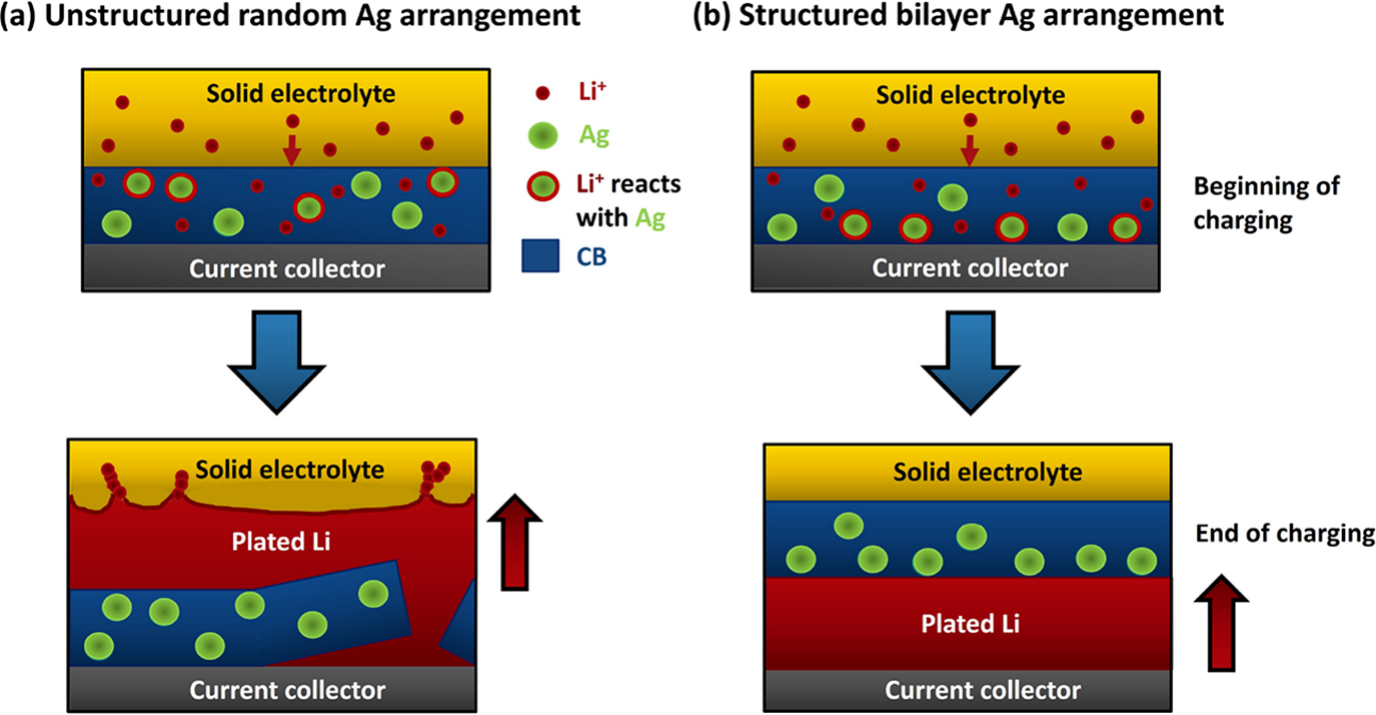
Schematic and idealized illustration of the Li plating mechanism upon charging for different Ag/CB interlayer arrangements. The plating mechanism for (a) unstructured and (b) structured Ag/CB interlayers.

There will be a tendency to form Li close to the current collector. For Ag enrichment at the current collector, the forming Li metal on the current collector progressively “pushes” the interlayer away from the current collector. For the unstructured interlayer, metallic Li again precipitates from the CB but, consistent with the random Ag distribution, is more distributed within the interlayer, as well as on the current collector. On subsequent cycling, there is an increased tendency for isolated or “dead” regions of Li far and disconnected from the current collector to develop, leading to a more marked capacity fading and progressive reduction in round-trip efficiency.^54^ The cooperative physical rearrangement of the structured Ag/CB interlayer is facilitated by the compliant nature of the “spongy” porous CB-rich region.^55–57^ If the interlayer is displaced toward the separator, it also forms a barrier that prevents Li from touching the LPS separator and thus making the nucleation of Li at separator surface flaws to form incipient dendrites, shown in Figure 5a, less likely.^58–62^

## Conclusions

In summary, an Ag/CB interlayer with increased Ag concentration toward the current collector was fabricated successfully using spray printing. Although there was unavoidable Ag agglomeration, both structured and unstructured Ag/CB interlayers facilitated metallic Li formation on the first charge cycle at a reduced overpotential. On subsequent cycling, the structured Ag bilayer arrangement was superior and delivered an initial discharge capacity of >190 mAh/g and slower capacity degradation during cycling, at a high Coulombic efficiency of >98% after 100 cycles. The structured interlayer enabled the formation of a relatively uniform Li plated layer and was identified by a combination of SEM, SIMS, EDS, and XPS. The study suggests there remain opportunities for design optimization of interlayers in SSB cells. Spray printing is a flexible platform to explore a wide range of interlayer designs with microscale composition control.

## Experimental Section

The detailed experimental methods regarding electrolyte and electrode preparation, cell assembly, electrochemical measurements, and characterization are elucidated in the Supporting Information.

## Supplementary Material



## Notes

Experimental section (including materials, spray printing of Ag/CB interlayers, laser cutting, characterization, cell preparation); laser diffraction analysis and SEM images of Ag and CB feedstock powders; HRTEM image and diffraction pattern of CB; XRD spectrum of CB; spatial distribution analysis of Ag particles in structured and unstructured Ag/CB interlayers; single-charging of three-electrode cells using structured and unstructured Ag/CB interlayers and an uncoated stainless-steel current collector; full-cell cycling using an uncoated stainless-steel current collector; full-cell cycling of structured/unstructured Ag/CB interlayers; SIMS of the Li/LPS bilayer reference; and XPS spectrum of the deposited Li anode; cross-sections of structured/unstructured Ag/CB interlayers after single charge (PDF)
