# Supplementary material for: Effect of Silver Particle Distribution in a Carbon Nanocomposite Interlayer on Lithium Plating in Anode-Free All-Solid-State Batteries
Source: ACS Appl Mater Interfaces. 2025 Jun 26;17(27):39089–96. doi: 10.1021/acsami.5c06550 (PMC12257451; doi:10.1021/acsami.5c06550)
Supplement: Supplementary file 1 [file am5c06550_si_001.pdf]

Supporting Information: The Effect of Silver Particle Distribution in a Carbon Nanocomposite Interlayer on Lithium Plating in Anode-Free All-Solid-State Batteries

Michael Metzler,[†,‡] Christopher Doerrer,[†,¶] Yige Sun,[†,‡] Guillaume Matthews,[†,‡] Enzo Liotti,[†] and Patrick S. Grant*[,†,‡]

†*Department of Materials, University of Oxford, Parks Road, Oxford, OX1 3PH, United Kingdom*

‡*The Faraday Institution, University of Oxford, Quad One, Becquerel Ave, Harwell Campus, Didcot, OX11 0RA, United Kingdom*

¶*Department of Mechanical and Industrial Engineering, University of Toronto, University of Toronto, 5 King's College Road, Toronto, ON M5S 3G8, Canada*

E-mail: patrick.grant@materials.ox.ac.uk

Experimental Methods

Spray Printing Ag/CB Interlayers

A solution of polyvinylidene fluoride (PVDF) in a mixture of 95% isopropyl alcohol (IPA) and 5% N-methyl-2-pyrrolidone (NMP) by volume was prepared. Ag nanoparticles (US Research Nanomaterials, Inc.; silver nanopowder, 50-80 nm) and carbon black (CB Asahi Carbon Co., Ltd.; F-200GS) were suspended in this solution at a CB: Ag ratio of 3:1 by

weight. The feedstock solution (277.9 mg particulate in 100 mL solvent) was stirred for at least 30 min and all suspensions were sonicated for 5 min (3 s on and 7 s off, 50% amplitude) using an ultrasonic horn sonicator (Qsonica 500) prior to spray printing. A reagent bottle containing the feedstock solution was connected to the spray printing system and the mixture continually stirred to avoid particle sedimentation. The feedstock solution was then spray printed onto a 10 μm thick stainless-steel foil that formed the anodic current collector in the subsequent solid-state cell. Spray printing was conducted in a custom-made spray tool (M-Solv, MSV-700G). The spray process was supplemented by an integrated vision system and a non-contact laser displacement height sensor that allowed for nozzle control and dimensional monitoring.

The feedstock solution was atomized by Ar gas in an industrial spray nozzle to create a myriad of suspension droplets that were deposited onto the heated stainless-steel foil where the solvent evaporated almost instantly and the PVDF binder thus rapidly reprecipitated to adhere the particulates to the substrate and to previously deposited layers. The metal foil was fixed by a vacuum chuck onto an Al hotplate which was kept at a constant temperature of 110 °C. One layer was formed through a zigzag motion of the spray nozzle in the x and y direction (10 mm pitch) in relation to the substrate at a constant distance in the z direction (190 mm). The total spray area was approximately 10×10 cm^2 and the 20 μm thick Ag/CB interlayers were typically formed from 18 layers.

To achieve a localized Ag concentration towards the current collector for the structured variant of the Ag/CB interlayer, 87.5 wt% of the total Ag was concentrated in the lower half of the interlayer, and the remaining 12.5 wt% content deposited in the upper half in two successive spray printing configurations.

Laser Cutting

The spray-printed Ag/CB interlayers were fixed to an Al holder plate and cut with a Needham NLase Desktop Pro laser cutter. The device applied a Q-switched, solid state, MOPA

laser that operates at a wavelength of 1064 $\pm$ 4nm and a peak laser power $>$ 10kW. The beam diameter was approximately 7 mm and had a beam divergence of $<$ 2 mrad. Laser cutting enabled cleaner cutting as shown in Figure S1 that compares discs obtained by laser cutting with those by mechanical punching.

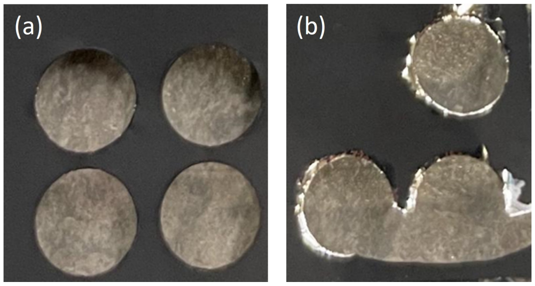

Figure S1: Comparison of Ag/CB interlayers on stainless steel foil obtained by (a) laser cutting that allowed cutting without tearing and delamination, and (b) mechanical punching.

Scanning Electron Microscope (SEM) and Energy-Dispersive X-Ray Spectroscopy (EDX)

Interlayers for SEM imaging were mounted on a Ti blade and cross-sectioned by broad beam Ar ion milling using a Gatan PECS II at a voltage of 8 kV. SEM analysis was performed with a Zeiss Merlin FEG-SEM equipped with an Oxford Instruments X-max EDX detector at an accelerating voltage of 10 kV.

Transmission Electron Microscope (TEM)

CB particles were dispersed in IPA and drop cast directly onto C-coated Cu grids. TEM analysis was performed using a Jeol 2100 TEM (LaB6 source) at an accelerating voltage of 200 kV.

X-Ray Diffraction (XRD)

XRD analysis of the CB was conducted using a PANalytical Empyrean diffractometer equipped with a K_α-Cu source. The spectrum was recorded at 40 kV and 40 mA.

X-ray Photoelectron Spectroscopy (XPS)

To characterize the composition of the deposited anode layer after charging via XPS, a cold-pressed three-electrode cell with a structured Ag/CB interlayer was charged at 1 mA/cm^2 for 1h (60 °C, 4 MPa stack pressure). The charged cell was disassembled, and the current collector upon which the anode was plated was removed for XPS-analysis. The deposited layer was etched to a depth of 25 nm to avoid measuring potential surface impurities. XPS was conducted with a Thermo Scientific K-Alpha XPS instrument using monochromated Al K_α line X-rays at 1486.6 eV. For a survey scan, a pass energy of 200 eV and a step size of 1 eV were applied. For individual scans, a step size of 0.1 eV was applied.

Plasma Focused Ion Beam (PFIB) with Secondary-Ion Mass Spectrometry (SIMS)

A Thermo-Fisher Scientific Helios G4 PFIB CXe DualBeam, equipped with a Xe^+ plasma focused-ion beam, was employed for cross-sectioning and polishing. To characterize the morphology of the electrode cross-section, electron-induced secondary electron (SE) images were acquired at 10 kV with a beam current of 1.6 nA. Additionally, utilizing the Xe^+ primary beam, ion-induced secondary electrons (iSEs) were observed at 30 kV with a current of 0.1 nA. For elemental mapping excluding Li, the spatial distributions of Ag, C, Fe, and S across the sectioned surface were acquired through EDS analysis using an Oxford EDS system with an Ultim Max 170 SDD detector. The data were captured and stored using Aztec software (version 4.1 SP1, Oxford Instruments), with a field of view (FoV) measuring 52.1 x 35.8 μm^2, comprised of 1024 x 704 pixels. To obtain the spatial distribution of $^7Li^+$ isotope at a spatial

resolution of 113 nm, SIMS was employed, utilizing a Hiden EQS SIMS detector. Imaging was conducted in the P-FIB with a field of view sized at 45.2 x 45.2 μm^2, containing 400 x 400 pixels. A Xe^+ beam operating at 30 keV and 1 nA was utilized to generate secondary ions. Hiden SIMS MAPPER software (version 2.0.0.5) was employed to map and record all SIMS data.

Cell Assembly

All electrochemical tests used a sulfide solid separator of approximately 1 mm thickness and 5 mm diameter. The cathode mixture was made by mixing sulfide SE, single crystal NMC (MSE Supplies, USA), and carbon nanofibers in a 40 mL ZrO_2 grinding bowl containing 55 g of 5 mm diameter ZrO_2 balls in a planetary mill (Fritsch Pulverisette 7) at 140 rpm for 30 min. A sulfide SE pellet from typically 30 mg powder was pressed uniaxially in a PEEK mold (5 mm diameter) at 250 MPa at room temperature. Subsequently, 4 mg of cathode powder mixture and then the Ag/CB coated current collector were inserted, and the arrangement uniaxially pressed against the LPS powder at 500 MPa. Finally, a 15 μm thick Al foil was added as current collector for the cathode.

Li electrodes for half-cells were prepared by rolling Li foil (Sigma-Aldrich, UK) in a pouch to approximately 150 μm. Circular electrodes of 3 mm and 1 mm diameter were punched out with a hand-held stainless steel cutting tool (EMS, Germany). A 30 μm thick Cu foil was used as current collector for the Li electrodes.

Electrochemical test data was evaluated using "Data Analysis" software (Batalyse, Germany).

Laser Diffractometry

To measure nanoparticle size distribution, feedstock Ag and CB were dispersed in IPA. Measurements were conducted at 15 °C using a NanoSight Nano Tracking Analysis laser diffraction analyzer with a Blue405 laser and an sCMOS camera capturing at 25 FPS. NTA

3.4 Build 3.4.4 software was used to obtain particle size distribution data.

Ag and CB Agglomeration

Laser diffraction analysis of the feedstock Ag and CB shown in Figures S2a and b indicated the presence of agglomerates in the pristine material, which were also visible for the Ag powder in the SEM, as shown in Figure S2c. These agglomerates proved difficult to break up entirely and were retained into the Ag/CB interlayers shown in Figure 2. The agglomeration

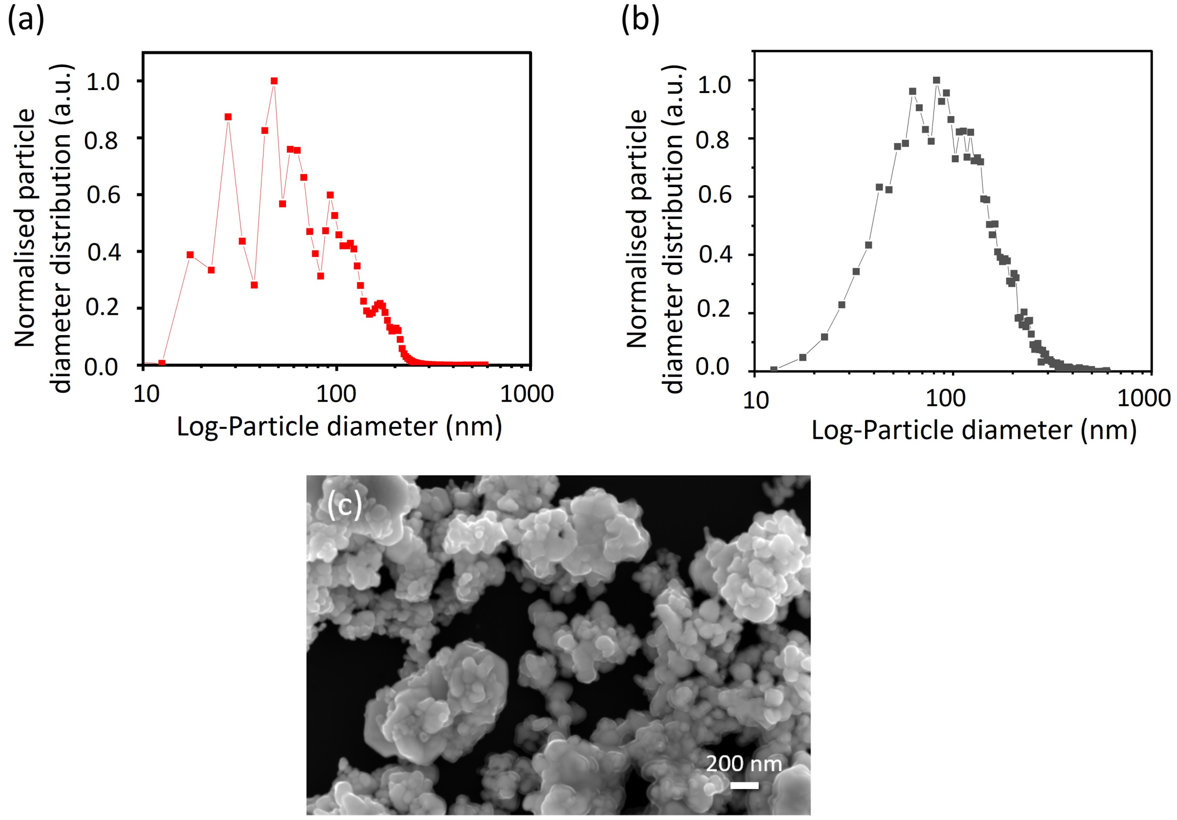

Figure S2: Analysis of the Ag and CB feedstock powders. Normalized particle size distribution obtained by laser diffraction in suspension of (a) Ag and (b) CB; and (c) SEM image of the as-supplied Ag powder.

reduced the overall surface area of Ag, likely limiting the reaction kinetics that underpin Li plating. An excessively uneven Ag distribution would likely lead to inhomogeneous Li plating. Overall, a better spacial distribution of Ag will be beneficial and boost Li plating

uniformity and CE further.

Figure S3(a) shows a high magnification SEM image of the pristine CB powder also with a strong tendency to agglomerate into extended networks. Figure S3(b) shows a high-resolution transmission electron microscopy (HRTEM) image of the CB agglomerates and Figure S3(c) shows a corresponding diffraction pattern comprising blurred rings suggesting the CB was largely amorphous, and consistent with previous reports.[S1,S2] Figure S3(d) shows

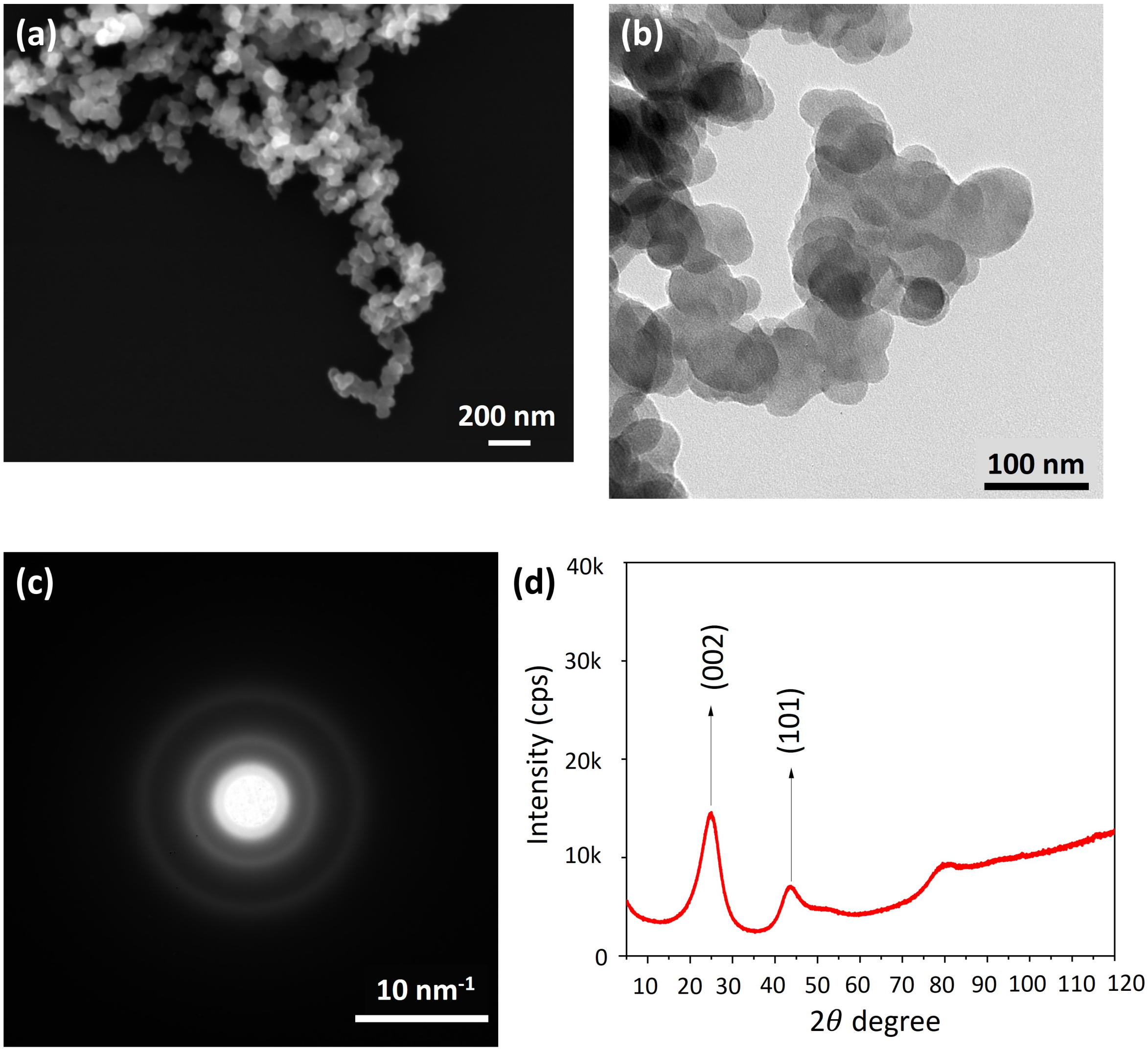

Figure S3: The microstructure of pristine CB. (a) SEM, and (b) HRTEM images; (c) corresponding HRTEM diffraction pattern: (d) XRD spectrum of CB.

the X-ray diffraction (XRD) spectrum from the CB particulates with a broad underlying peak again suggesting a predominantly amorphous structure, but also with superimposed broad

peaks at approximately 24.0° and 43.3°, which were ascribed to (002) and (101) planes of disordered carbon (JCPDS card No. 34-0567, DOI: 10.1002/adfm.201604356). This may indicate a turbostratic carbon structure comprising a largely amorphous "matrix" with fine-scale regions of crystalline carbon.[S3]

Ag Spatial Distribution

In order to quantify differences in the Ag distribution in the Ag/CB interlayers, the cumulative Ag area fraction of the SEM backscattered electron images shown in Figure 2 was measured along the vertical, through-thickness direction. The local Ag fraction was obtained by segmenting the Ag particles within the SEM images by global intensity thresholding (Otsu's method) and cumulatively summing the segmented area, line by line of pixels through the interlayer, followed by normalisation by the total Ag area. The spatial distribution of Ag in the structured interlayer shown in Figure S4(a) illustrates the successful Ag concentration towards the current collector compared with the unstructured interlayer shown in Figure S4(b).

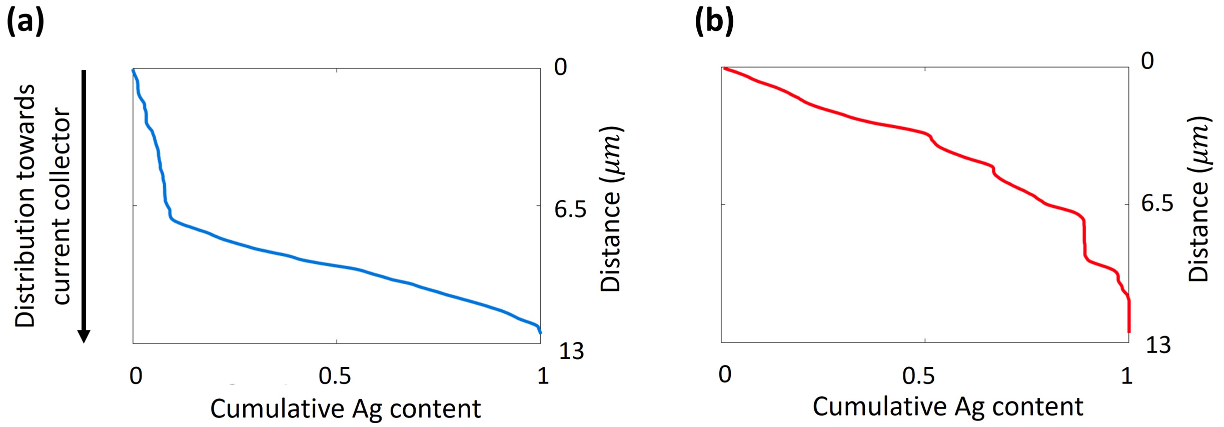

Figure S4: Spatial distribution analysis of Ag particles in (a) structured and (b) unstructured Ag/CB interlayers after uniaxial cold pressing at 500 MPa.

Electrochemical Characterization of Ag/CB Interlayers

Figure S5 compares the voltage versus Li^+/Li at the counter electrode as a function of time for the first charge cycle of three-electrode cells at charge currents of 1 mA/cm^2 and 0.5 mA/cm^2 respectively, up to a capacity of 1 mAh/cm^2. While structured and unstructured interlayers significantly lowered the overpotential magnitude to initiate plating, there was no resolvable, reproducible overpotential difference. There were no significant differences in the profiles between structured and unstructured cells, i.e. intercalation and plating dynamics were broadly similar for the first charge.

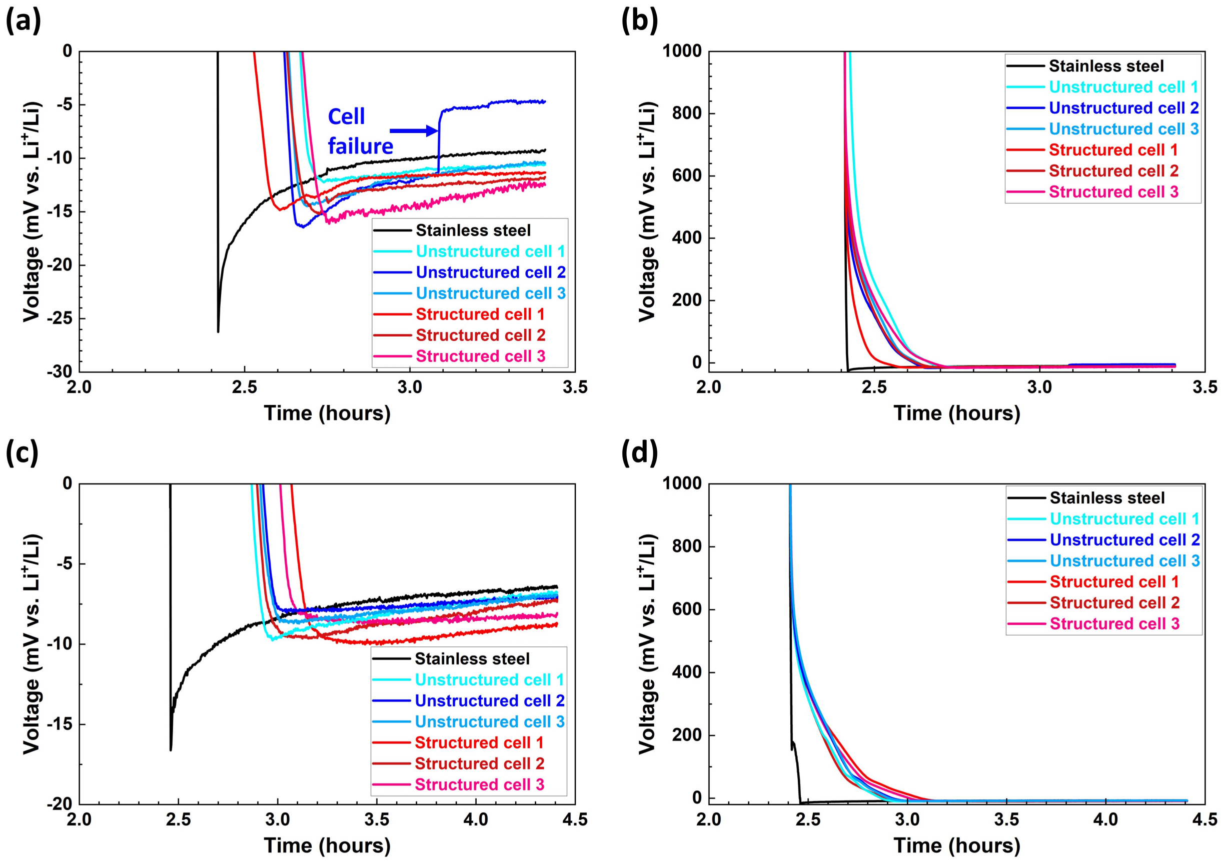

Figure S5: Single-charging of three-electrode cells using a stainless-steel current collector and structured and unstructured Ag/CB interlayers at (a)/(b) 1 mA/cm^2 and (c)/(d) 0.5 mA/cm^2, to a capacity of 1 mAh/cm^2.

Figures S6(a) and (b) show the reproducibility of Ag/CB structuring on charge and discharge capacity, respectively, over the first three cycles. In both cases, and despite cell-to-cell variations, the structured interlayer supported a higher achieved capacity.

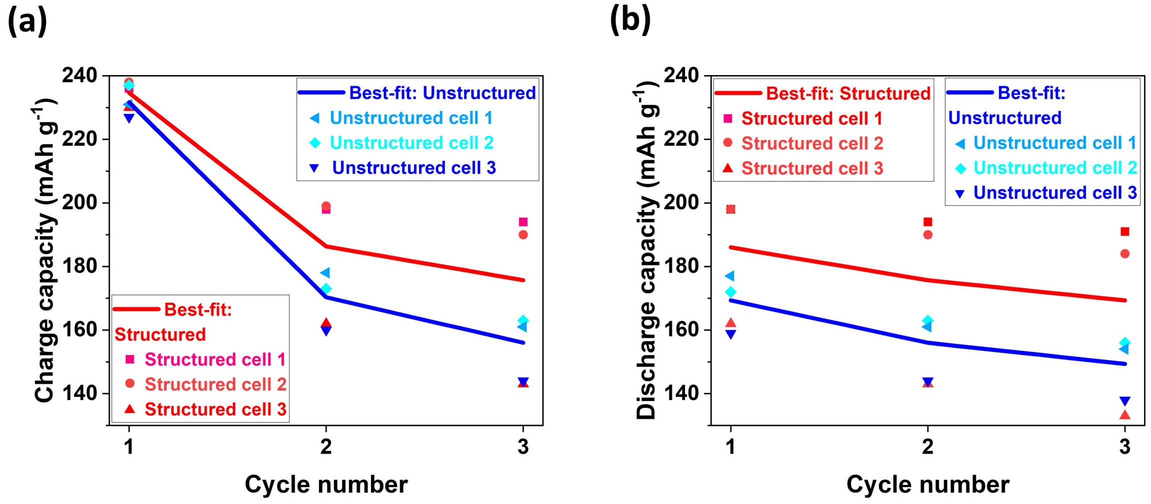

Figure S6: (a) Charge and (b) discharge capacities for three full-cells with structured and unstructured Ag/CB interlayers each.

Figure S7 shows the voltage-charge response of an uncoated stainless-steel current collector in a full-cell arrangement that did not yield a recognisable cycling response.

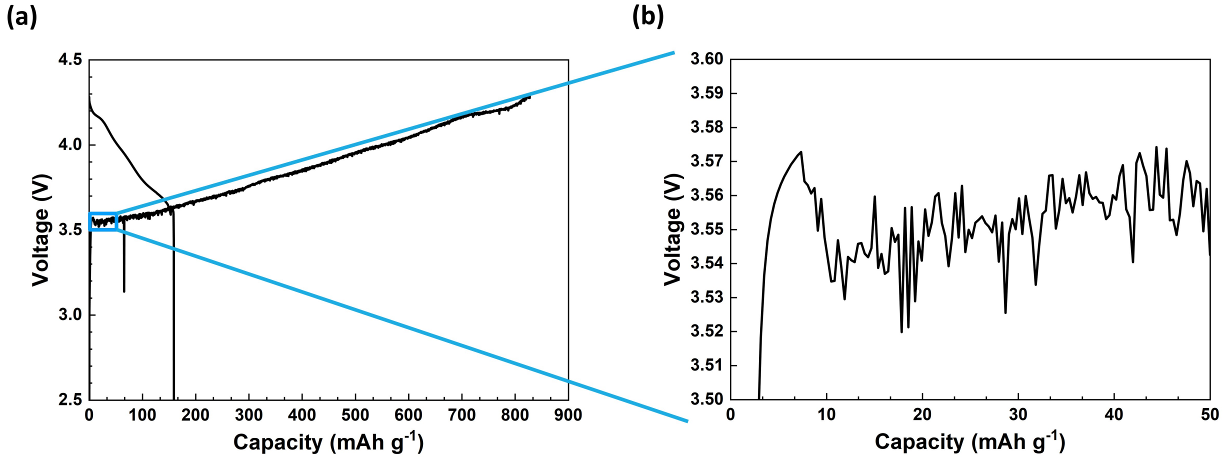

Figure S7: (a) Full-cell cycling test using an uncoated stainless-steel current collector at a charge rate of 1 mA/cm^2, discharge rate of 0.5 mA/cm^2, and stack pressure of 20 MPa; and (b) zoomed in charging showing signs of significant instability.

[Figure S8](#) shows full cell charge capacity during cycling at a lower discharge rate of 0.2 mA/cm^2 and higher stack pressure of 20 MPa than used in Figure 3. The structured Ag/CB interlayer improved charge capacity retention from 40% to 45%.

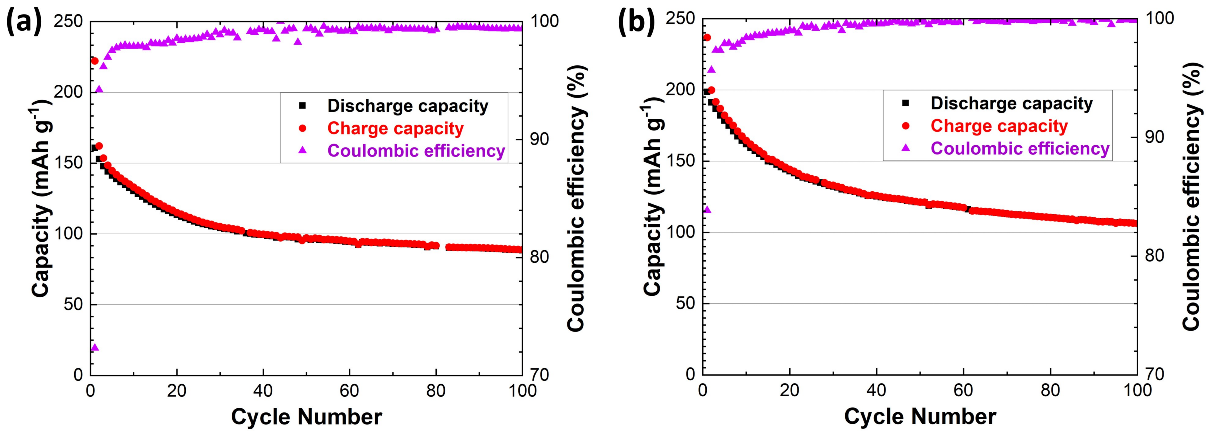

Figure S8: Cycling of full-cell setup at 1 mA/cm^2 charge rate and 0.2 mA/cm^2 discharge rate at a stack pressure of 20 MPa for (a) unstructured and (b) structured Ag/CB interlayers.

Impedance analysis of full cells after charge/discharge cycling at 1/0.5 mA/cm^2 and 4 MPa and after 1, 5, and 10 cycles is shown in the Nyquist plots in Figure S9(a). A frequency range of 10 mHz – 10 kHz with a nominal AC voltage of 10 mV was applied. The best-fit to the structured interlayer first cycle data using a typical equivalent circuit model (EC-Lab software) is shown in Figure S9(b). The data revealed an ionic conductivity of $\sim$ 5 mS/cm for both cells and no resolvable difference between the Ag/CB arrangements or in their impedance evolution up to 10 cycles.

Figure S10(a) shows rate capability tests of full cells at current densities of 1 mA/cm^2 and 2 mA/cm^2 for 5 cycles, respectively, at a stack pressure of 2 MPa. Increased current density resulted in faster capacity degradation and cell failure occurred at 3 mA/cm^2 for both structured and unstructured interlayers. Figures S10(b) and (c) compare the voltage versus Li^+/Li at the counter electrode as a function of time for the first charge cycle of hot-pressed three-electrode cells at charge currents of 2.5, 3, and 3.5 mA/cm^2 respectively, up to a capacity of 5 mAh/cm^2. Stable charging of structured and unstructured interlayers

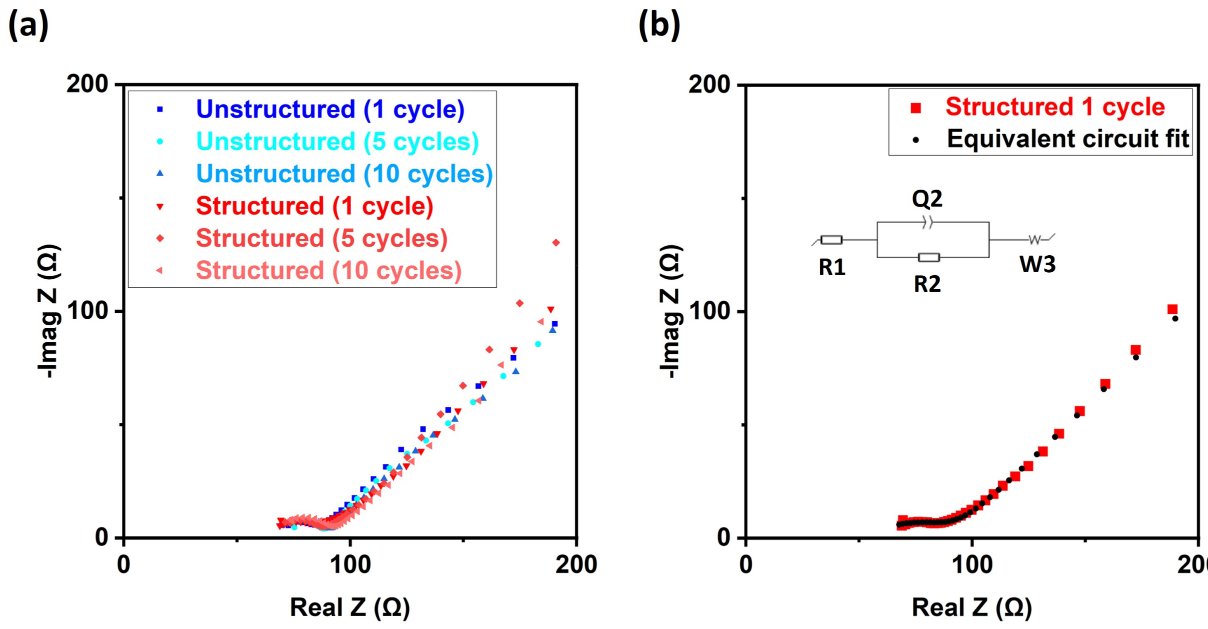

Figure S9: (a) Nyquist plots of structured and unstructured interlayers after 1, 5, and 10 cycles; (b) equivalent circuit fit to the structured interlayer after 1 cycle. R1 = 53.46 Ω, R2 = 40.54 Ω, Q2 = 0.69 × 10^{-3} $F.s^{(\alpha-1)}$.

took place at 2.5 mA/cm^2. The unstructured interlayer displayed a more unstable charging curve at 3 mA/cm^2 and cell failure occurred in both cases at 3.5 mA/cm^2.

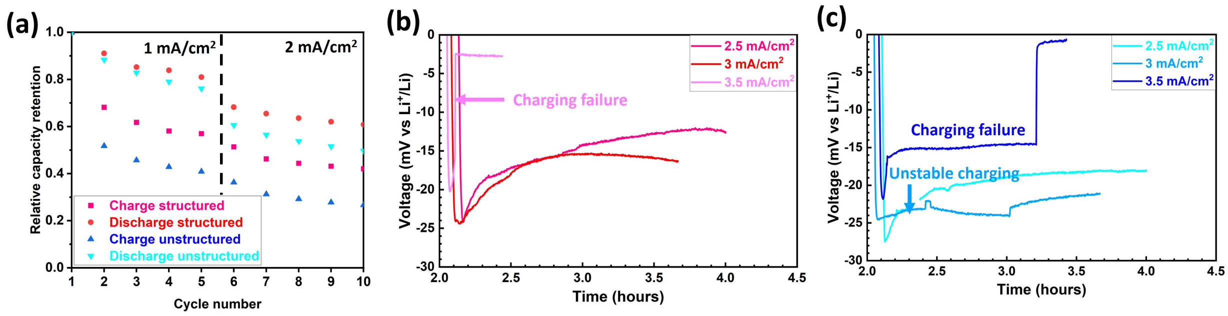

Figure S10: Rate capability tests of (a) full-cell setup at 1 and 2 mA/cm^2 charge and discharge rate at a stack pressure of 2 MPa of unstructured and structured Ag/CB interlayers; (b) rate capability test of structured three-electrode cells; (c) rate capability test of unstructured three-electrode cells.

SIMS Analysis of a Li/LPS bilayer reference

A piece of high-purity Li was pressed uniaxially onto a dense LPS pellet at 500 MPa at room temperature to form a Li/LPS bilayer reference for SIMS analysis. The Li/LPS Li intensity ratios for the charged Ag/CB cell and the reference bilayer are shown in Figure S11. The Li/LPS Li intensity ratios were 36:100 and 38:100, respectively, confirming the presence of plated Li between the stainless steel (SS) current collector and the sprayed Ag/CB structured interlayer. The significant drop in Li/LPS signal intensity ratio for the Ag/CB region in the plated cell relative to the adjacent Li-containing sections indicated a low concentration of Li in CB-rich parts of the interlayer. This suggested that the Ag/CB interlayer functioned principally as a catalyst for Li plating rather than storing significant Li concentrations itself. Post-mortem analysis of the plated Li layer showed no strong evidence for distinct Li-Ag phases at the resolution of the instrumentation available. However, some of the EDS maps (e.g. Figure S-13(g)) showed an Ag signal from the Li layer, which may be an anomaly of Ag redeposition during specimen preparation, or evidence of a small amount of Ag dissolved in Li solid solution.

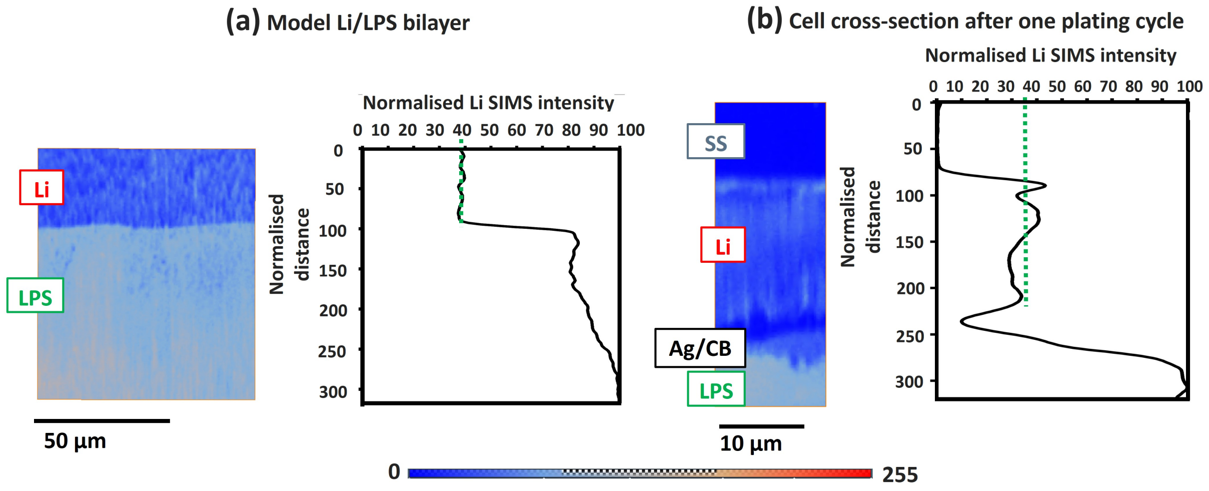

Figure S11: Cross-section Li SIMS maps of (a) the model Li/LPS reference bilayer, and (b) the anodic current collector region of a cell with a structured Ag/CB interlayer after one plating cycle at 2.5 mA/cm^2. In each case the Li SIMS intensity from the LPS region was set to 100 and the Li SIMS intensity from other regions scaled accordingly. The green dotted line indicates the intensity in (a) Li foil and (b) the nominal Li plated layer.

XPS Analysis of Deposited Li Anode

Figure S12 shows the XPS spectrum from the deposited layer at the anode from a single-charged cell at 1 mA/cm^2 with a structured Ag/CB interlayer. The strong oxidised Li metal signal and no other significant candidate peaks confirmed Li metal plating.

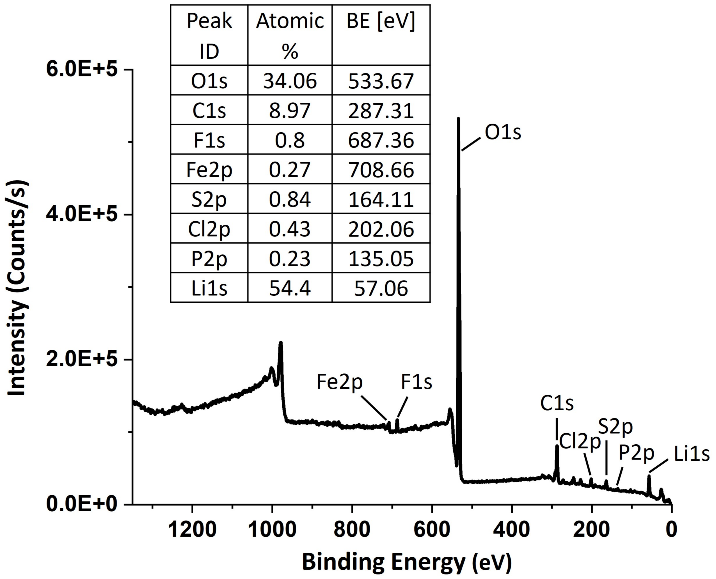

Peak ID	Atomic %	BE [eV]
O1s	34.06	533.67
C1s	8.97	287.31
F1s	0.8	687.36
Fe2p	0.27	708.66
S2p	0.84	164.11
Cl2p	0.43	202.06
P2p	0.23	135.05
Li1s	54.4	57.06

Figure S12: XPS spectrum and peak table of the layer deposited at the anode after one cycle at 1 mA/cm^2. The detected signals were attributed as follows: O – oxidized Li; C – interlayer, oxidized Li; P, S, and Cl - solid electrolyte; Li – solid electrolyte and oxidised Li at the anode; F - PVDF binder in interlayer, and Fe - current collector.

Thickness Variations of Li Anode Deposition

The expected plated Li thickness after charging was estimated as follows:

Transferred material per area = (theoretical capacity of Li: 3482 mAh/g) × (applied charge of 2.5 mA/cm^2 for 2 hours) = 0.001436 g/cm^2

The diameter of Ag/CB working electrode of 2 mm had an area of 0.03141 cm^2.

Therefore, the transferred mass of Li = 0.001436 g/cm^2 × 0.03141 cm^2 = 4.51 × 10^{-5} g

Volume = transferred mass (4.51 × 10^{-5} g)/density of Li (0.534 g/cm^3) = 8.44792 × 10^{-5} cm^3

Theoretical thickness of the Li layer = volume/area ≈ 27 μm

Figures S13(a) and (e) show the Li deposition via structured/unstructured interlayers, respectively, after a single plating cycle at a current density of 2.5 mA/cm^2 for 2h at a wider field of view than Figure 4. Figures S13(b) to (d) show EDS maps of Fe, Ag, and C, respectively, for the unstructured interlayer, indicating a tendency for Ag/CB interlayer retention on the current collector, rather than displacement of the interlayer and consistent Li plating on the current collector. Figures S13(f) to (h) show EDS maps of Fe, Ag, and C, respectively, for Li plated via a structured interlayer compared with an unstructured interlayer. Compared with an unstructured interlayer, there was again an increased tendency of the Ag/CB interlayer to displace towards the separator and more consistent plating of the Li directly onto the current collector. While there were local variations in the plated Li thickness (20-25 μm), the plated Li thickness was approximately similar to the estimated thickness of 27 μm.

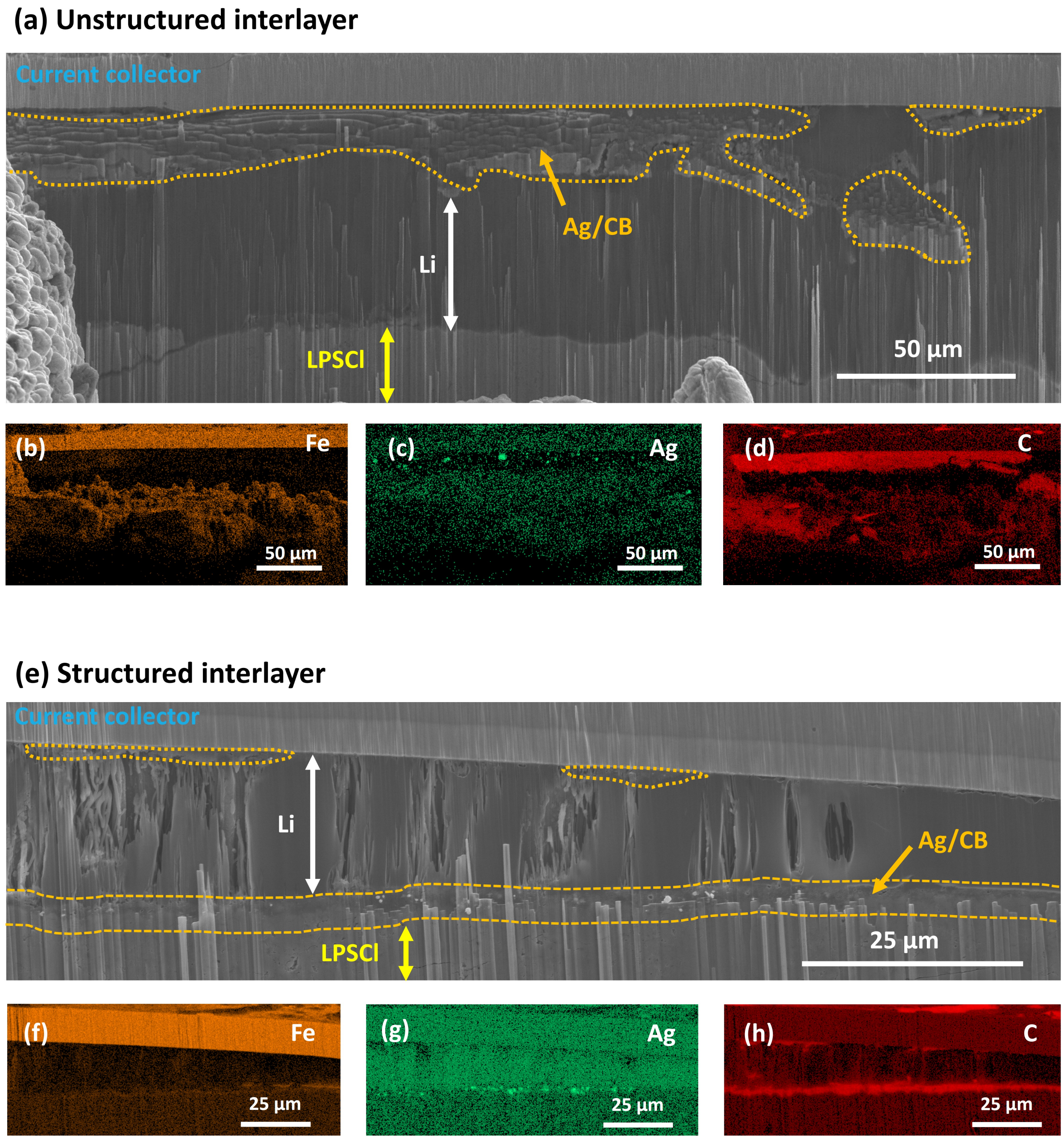

Figure S13: A cross-section of the stainless steel current collector, plated Li layer, Ag/CB interlayer and LPS separator region for a single charge at 2.5 mA/cm^2. (a) SEM image of the unstructured Ag/CB arrangement; (b) to (d) corresponding EDS maps for Fe, Ag, and C respectively; (e) SEM image of the structured Ag/CB arrangement; (f) to (h) corresponding EDS maps for Fe, Ag, and C respectively.

Figure S14 shows an SEM image and corresponding Fe, Ag, and C EDS maps of a structured interlayer after a single plating cycle at a slightly lower current density of 2

mA/cm^2 for 2.5 h, again showing an approximately 20 μm Li layer plated onto the current collector.

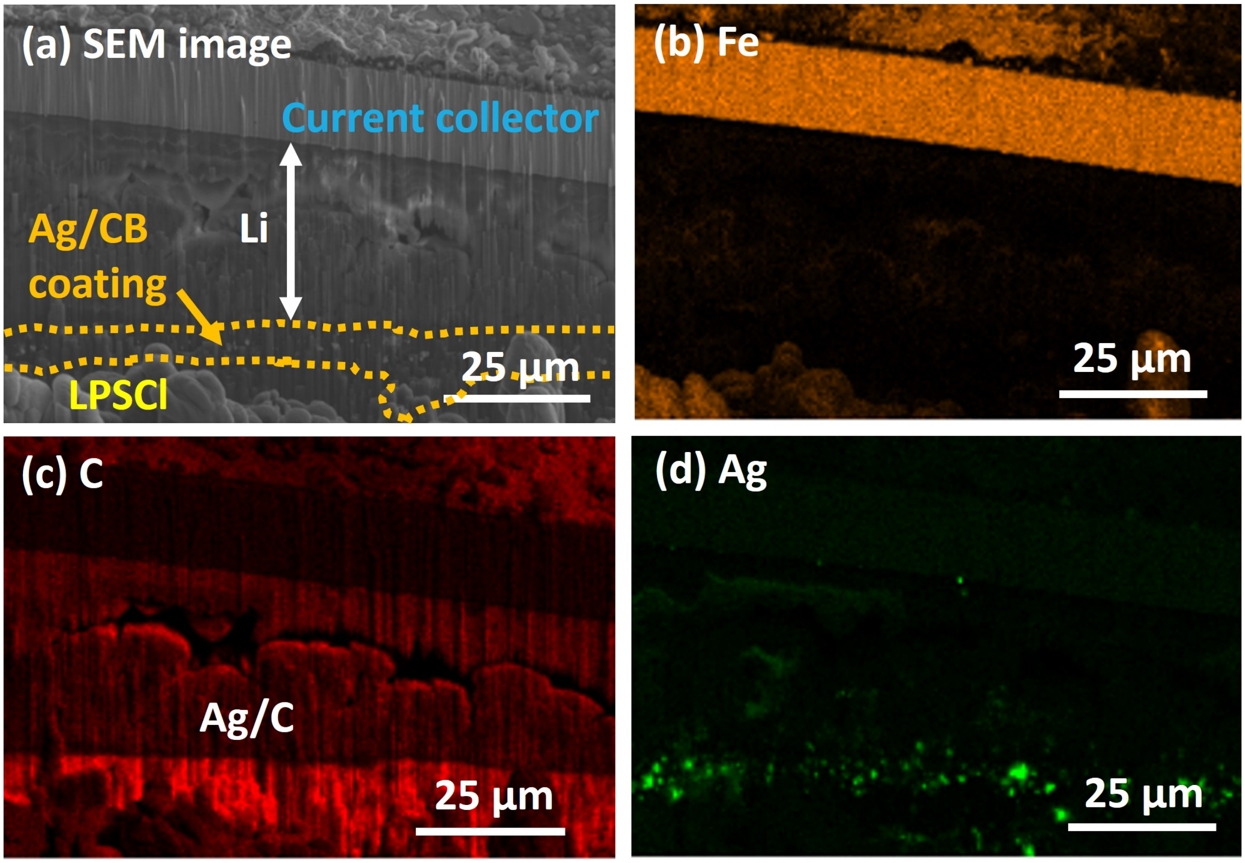

Figure S14: A cross-section of the stainless steel current collector, deposited layer, structured Ag/CB interlayer and LPS separator region for a single charge at 2 mA/cm^2. (a) SEM image of the structured Ag/CB; (b) to (d) corresponding EDS maps for Fe, C, and Ag respectively.

References

(S1) Yoo, S. J.; Kim, C.-Y.; Shin, J. W.; Lee, S.-G.; Jeong, J.-M.; Kim, Y.-J.; Lee, S.-H.; Kim, J.-G. Characterization of an Amorphous Carbon Film Covering a Mo Grid During in situ Heating TEM Study. *Mater. Charact.* **2013**, *78*, 31–36.

(S2) Kwiecińska, B.; Pusz, S.; Valentine, B. Application of Electron Microscopy TEM and SEM for Analysis of Coals, Organic-Rich Shales and Carbonaceous Matter. *Int. J. Coal Geol.* **2019**, *211, No. 103203*.

(S3) Ruz, P.; Banerjee, S.; Pandey, M.; Sudarsan, V.; Sastry, P. U.; Kshirsagar, R. J. Structural Evolution of Turbostratic Carbon: Implications in H2 Storage. *Solid State Sci.* **2016**, *62*, 105–111.
